# Acute effects of cannabigerol on anxiety, stress, and mood: a double-blind, placebo-controlled, crossover, field trial

**DOI:** 10.1038/s41598-024-66879-0

**Published:** 2024-07-13

**Authors:** Carrie Cuttler, Amanda Stueber, Ziva D. Cooper, Ethan Russo

**Affiliations:** 1 Department of Psychology, Washington State University, PO Box 644820, Pullman, WA 99164-4820 USA; 2 UCLA Center for Cannabis and Cannabinoids, Jane and Terry Semel Institute for Neuroscience and Human Behavior, Department of Psychiatry and Biobehavioral Sciences, Department of Anesthesiology and Perioperative Medicine, University of California, Los Angeles David Geffen School of Medicine, Los Angeles, USA; 3 CReDO Science, Vashon, WA USA

**Keywords:** Cannabigerol, Anxiety, Stress, Impairment, Intoxication, Memory, Psychology, Human behaviour

## Abstract

Cannabigerol (CBG) is a phytocannabinoid increasing in popularity, with preclinical research indicating it has anxiolytic and antidepressant effects. However, there are no published clinical trials to corroborate these findings in humans. The primary objective of this study was to examine acute effects of CBG on anxiety, stress, and mood. Secondary objectives were to examine whether CBG produces subjective drug effects or motor and cognitive impairments. A double-blind, placebo-controlled cross-over field trial was conducted with 34 healthy adult participants. Participants completed two sessions (with a one-week washout period) via Zoom. In each, they provided ratings of anxiety, stress, mood, and subjective drug effects prior to double-blind administration of 20 mg hemp-derived CBG or placebo tincture (T0). These ratings were collected again after participants ingested the product and completed an online survey (T1), the Trier Social Stress Test (T2), a verbal memory test and the DRUID impairment app (T3). Relative to placebo, there was a significant main effect of CBG on overall reductions in anxiety as well as reductions in stress at T1. CBG also enhanced verbal memory relative to placebo. There was no evidence of subjective drug effects or impairment. CBG may represent a novel option to reduce stress and anxiety in healthy adults.

## Introduction

The rapid proliferation of the new legal cannabis market has instigated producers to cultivate an array of novel products to satisfy consumers’ growing interest. While products high in delta-9-tetrahydrocannabinol (THC) continue to saturate the market^1^, a growing body of consumers are seeking alternative non-intoxicating products carrying the promise of easing what ails them. Producers are responding to this demand by isolating various minor phytocannabinoids and terpenes and marketing them with bold, but largely unsubstantiated, claims of their therapeutic potential. While cannabidiol (CBD) continues to be the dominant non-intoxicating cannabinoid of interest to both consumers and researchers, cannabigerol (CBG) has rapidly increased in popularity^2^.

CBG is a minor phytocannabinoid and its acidic form (CBGA) is often referred to as “the mother of all cannabinoids” as it is a precursor to numerous other phytocannabinoids, including THC, CBD, and cannabichromene (CBC). CBG received little research interest initially, due in part to the overwhelming focus on the effects of THC and CBD. Subsequent pre-clinical investigations involving the administration of CBG to animals, however, have demonstrated a broad spectrum of potential therapeutic effects including potent antibiotic^3^ and antifungal activity^4^. CBG also appears to have anti-hypertensive effects^5^, it reduces intra-ocular pressure^6^ and keratinocytes in a psoriasis model^7^, it has possible efficacy in inflammatory bowel disease^8^ and it may have analgesic effects^9^. Moreover, CBG has been demonstrated to have antidepressant-like effects in a rodent tail suspension model^10^ while lacking cannabimimetic effects indictive of THC^10,11^.

In stark contrast to the impressive body of preclinical research, there has been a dearth of research examining effects of CBG on humans. To help fill this gap, we recently published a study in which 127 experienced CBG users were surveyed on their use of CBG-dominant products, including their use patterns, the perceived therapeutic effects of CBG, as well as its potential side effects^12^. Participants most frequently reported using CBG to manage anxiety (attested to by 51% of the sample), chronic pain (41%), depression (33%), and insomnia (31%). Moreover, most of the sample indicated that CBG was more effective than conventional medications for treating anxiety (78%), chronic pain (74%), depression (80%), and insomnia (73%). Only a minority reported experiencing side effects such as dry eyes (9%), dry mouth (16.5%), sleepiness (15%), and increased appetite (12%). While provocative, these findings are limited by their retrospective, self-report, nature, and the use of preparations of varying CBG composition. As such, they require corroboration via double-blind, placebo-controlled clinical trials.

However, to date there are only two published clinical trials on the effects CBG in humans. One examined the effects of an oral formulation containing CBG (50 mg), CBD (30 mg), and the terpene beta caryophyllene (25 mg) on experimentally induced delayed onset muscle soreness^13^. The other study examined the impacts of dietary fat (low fat meal [< 5 g] vs. high fat meal [> 30 g]) and oral delivery systems (isolate vs. emulsification) on CBG pharmacokinetics^14^.

### Current study

The primary objective of the current study was to use a double-blind, placebo-controlled cross-over trial to investigate the acute effects of CBG on anxiety, stress, and mood. The secondary objectives were to assess subjective drug effects (intoxication, drug effect, drug liking), potential side effects (dry eyes, dry mouth, sleepiness, appetite, racing heart/heart palpitations) as well as to determine whether CBG produces motor or cognitive impairments. We hypothesized that CBG would decrease anxiety, decrease stress, and elevate mood. Further, we hypothesized that ratings of subjective drug effects would be low and that CBG would not produce motor or cognitive impairments.

## Methods

### Design

A double-blind, placebo-controlled cross-over field trial was used to address our primary and secondary objectives. There was a one-week wash out period between the two testing sessions. Orally administered CBG has been demonstrated to have a half-life of 2–6 h. in rodents and dogs^15,16^, thus this 1-week washout period would be ample to clear any residual drug effects. We used a 1:1 allocation ratio and potential carry over effects were controlled for by adding order of drug/placebo administration as a covariate in the statistical analyses.

Data collection commenced March 2022 and was completed November 2023. The study was pre-registered on ClinicalTrials.gov [NCT05257044] (25/02/2022) and was approved by the Washington State University (WSU) Institutional Review Board (IRB). All participants provided informed consent and the research was conducted in accordance with the Declaration of Helsinki. The trial was conducted remotely via Zoom from The Health & Cognition (THC) lab at WSU. The use of Zoom helped enhance feasibility and generalizability by allowing us to recruit a more diverse sample of participants residing at various locations across the state rather than limiting recruitment to our small university town.

### Participants

#### Power

Results of an a priori power analysis using G-Power indicated that a sample size of 34 would be required to achieve power of 0.80 to detect medium-sized effects (i.e., $d$ = 0.50) with alpha set at 0.05.

#### Inclusion/exclusion criteria

Participants were required to be at least 21 years of age, reside in Washington State, have a smartphone, and have access to a computer with a webcam connected to stable internet in a private environment. They also had to be fluent in English and able to see, hear, and read. Participants could not have any chronic neurological disorders, head injuries involving a loss of consciousness for more than 10 min, or have a diagnosed intellectual disorder, psychotic disorder, autism spectrum disorder, or bipolar disorder. Participants were also excluded if they were pregnant or breastfeeding, or if they reported any illicit drug use (except cannabis) in the past 2 months. To reduce risks of adverse reactions, participants had to report prior experience with cannabis-based products (i.e., ≥ 10 lifetime uses, use in the past month) and report no serious prior adverse reactions to CBG (e.g., panic attacks, psychosis). Finally, participants had to agree to abstain from using products containing cannabis or CBG for a minimum of 24 h prior to the testing session. Initially, participants were required to report no heavy alcohol use, no illicit drug use in the past 3 months (rather than 2 months) and prior experience using CBG (rather than with cannabis-based products). They were also initially required to abstain from use of cannabis and CBG for a minimum of 72 h prior to the testing session. These four criteria were changed 5 months into the trial to bolster recruitment and minimize cannabis withdrawal symptoms as only two participants were enrolled and tested prior to this change.

### Materials

#### Study drug

The CBG tincture was composed of 10 mg/ml CBG, 0.89 mg/ml CBGA, 0.35 mg/ml β-caryophyllene (0.51 mg/ml total terpenoids). It contained < 0.001 mg/g THC and CBD and was derived from a CBG-dominant hemp plant containing less than the 0.3% THC limit imposed in the U.S. Agricultural Improvement Act of 2018. It was extracted from inflorescences in 190 proof (95%) ethanol. It was donated by Shango Los from a single batch of material with a certificate of analysis provided from Analytical 360 Analysis Laboratory (Yakima, WA; see Certificate of Analysis in Supplemental Materials S1). Single 3 ml amber vials were prepared with this material containing 2 ml each representing a dose of 20 mg of CBG. Chartreuse liqueur (55% ethanol by volume) prepared by Les Pères Chartreux, France 1 ml, diluted with 1 ml filtered water was employed as the placebo as it provided a reasonable match for the green color and herbal/ethanol taste of the CBG tincture. Opaque vials were labeled with color coded tabs (Blue = CBG, Yellow = Placebo) in double-blind fashion and mailed directly to study participants. To dilute the taste, participants were instructed to mix the contents of the vial in a small glass of water prior to ingestion.

#### Online questionnaires

##### Demographic questionnaire

Participants responded to a small series of items designed to assess demographic characteristics. Specifically, they were asked to provide information pertaining to their gender, age, ethnicity, education, personal income, work status, and marital status.

##### Daily sessions, frequency, age of onset, and quantity of cannabis use inventory (DFAQ-CU)

The DFAQ-CU is a 41-item inventory, with 24 core items that assess frequency, age of onset, and quantity of cannabis flower, quantity of cannabis concentrates, and quantity of edibles typically used^17^. The remaining items measure other aspects of cannabis use not commonly measured by other cannabis use scales (e.g., forms of cannabis; methods of administration; use for medical, recreational, or combined purposes). The subscales have shown acceptable internal consistency as well as good predictive, convergent, and discriminant validity^17^. This inventory was simply used to describe the cannabis using patterns of participants.

##### State-trait anxiety inventory (STAI)

The STAI is an inventory for measuring state and trait anxiety^18^. The inventory contains two parts, one containing 20 items designed to assess state anxiety and another containing 20 items designed to assess trait anxiety. The state anxiety section assesses how individuals feel in the moment. The trait anxiety section assesses how individuals generally feel. We computed state and trait anxiety scores according to Spielberger et al.^18^. Possible scores on each section range from 20 to 80, with higher scores reflecting more anxiety. The inventory has been demonstrated to have high reliability coefficients^18^. In the present study, Cronbach’s alpha exceeded 0.90 for trait anxiety and state anxiety at baseline as well as for the repeated assessments of state anxiety in both conditions.

##### Depression anxiety stress scales (DASS-21)

Levels of depression, anxiety, and stress were assessed using the DASS-21, which is a self-report inventory designed to measure these different, but related constructs^19^. Each of the three subscales contained seven statements and participants indicate how much each statement has applied to them in the past week. Scores for each subscale were computed by summing the scores for each subscale and multiplying the sum by two, with higher scores representing stronger endorsement of that dimension of distress. The DASS-21 and its subscales have been shown to possess good psychometric properties^20,21^. In the present study, Cronbach’s alpha for depression was 0.92 in the CBG condition and 0.94 in the placebo condition, for anxiety it was 0.75 in the CBG condition and 0.69 in the placebo condition, and for stress it was 0.90 in the CBG condition and 0.91 in the placebo condition.

#### Subjective state ratings

Participants rated their subjective levels of anxiety and stress using a 0 (not at all) to 10 (extremely) visual analogue scales (VAS), and they rated their subjective mood using a 0 (extremely negative) to 10 (extremely positive) VAS. Cronbach’s alpha was 0.94 for anxiety ratings in both conditions, it was 0.90 for stress in the CBG condition and 0.92 for stress in the placebo condition, and it was 0.88 for mood in the CBG condition and 0.90 for mood in the placebo condition.

#### Subjective drug effect ratings

Participants rated their levels of intoxication using 0 (not at all intoxicated) to 10 (extremely intoxicated) VAS, the level of drug effects they were experiencing using 0 (none) to 10 (a lot) VAS, and their liking of the drug effects using 0 (dislike very much), 5 (neutral) to 10 (like very much) VAS. Similarly, they rated their level of dry eyes, dry mouth, sleepiness, appetite, and racing heart/heart palpitations using 0 (none) to 10 (extremely) VAS. Cronbach’s alpha exceeded 0.90 for ratings of dry eyes, dry mouth, sleepiness, and appetite in both conditions and drug effect ratings in the placebo condition; it exceeded 0.80 for intoxication ratings in the CBG condition; it exceeded 0.70 for heart palpitations in both conditions, drug effect ratings in the CBG condition and drug liking ratings in the placebo condition. Finally, Cronbach’s alpha was 0.66 for drug liking ratings in the CBG condition and it was 0.68 for intoxication ratings in the placebo condition.

#### Online trier social stress test

Participants completed an online version of the Trier Social Stress Test (TSST)^22^. Participants were instructed to mentally prepare a 5-min speech describing why they would be a good candidate for their ideal job. They were informed that the speech would be recorded and reviewed by a panel of judges trained in public speaking (although no such recording was made). They were sent to a Zoom breakout room for 10-min to prepare their speech. The research assistant (RA) put on a white lab coat while they were in the breakout room. Participants were brought back to the main Zoom room and were asked to deliver their speech to the RA who maintained a neutral expression. They were instructed to speak for the entire 5-min period and were prompted to continue if they stopped for more than 20 secs. Immediately upon completing the speech, they were told they would need to complete a 5-min math test. For this test they were instructed to sequentially subtract the number 13 from 1,022. Each time they made a mistake they were informed they made a mistake and were instructed to start over from 1,022. Prior research has found that this online version of the TSST significantly and robustly increases state anxiety, perceived stress, blood pressure, and heartrate compared to baseline^22^.

#### California verbal learning test-II

The standardized California Verbal Learning Test II (CVLT-II)^23^ was used to assess verbal free recall. Participants were asked to listen to and immediately recall a list of 16 words three times in a row (Trials 1–3). They were then asked to listen to and immediately recall a list of 16 new words (List B). Immediately following recall of List B, participants were required to recall the words from List A (Short Delay). Alternate forms of the lists were used in the first and second testing sessions to reduce practice effects. Cronbach’s alpha was 0.84 in the CBG condition and 0.91 in the placebo condition.

#### DRUID application

The benchmark version of the Driving under the influence of drugs (DRUID) mobile application contains four brief tasks (each under 45 s), completed on a smartphone, that measure cognitive and motor impairment. Past research has shown that the DRUID is a sensitive test of impairment that detected significant differences between placebo and both oral and vaporized THC^24^. Prior to completing the tests participants were instructed to stand up and to hold their phone in one hand and to tap the screen with their other hand. For the first task, circles and squares are briefly flashed on the screen and participants are instructed to tap the location on the screen where each circle flashed and to tap a white oval at the top of the screen whenever they see a square as quickly as possible. The instructions change mid-way through the task and participants are instructed to tap the location on the screen where each *square* flashed and to tap the white oval at the top of the screen whenever they see a *circle* as quickly as possible. For the second task, circles briefly appear on the screen, and participants are instructed to tap where they saw each circle as quickly as possible and to press STOP at the top of the screen when they have estimated that 30 secs have passed. For the third task, participants are instructed to keep their finger on a moving circle and count the number of squares that briefly appear on the screen. For the fourth task, participants are instructed to raise their left foot off the floor and balance on their right foot while holding their phone in their left hand for 15 s. Finally, they switch sides and raise their right foot off the floor while holding their phone in their right hand for 15 s. The app accesses data from the accelerometer in the smartphones to measure stability and balance during the latter task. The primary outcome measure is a global impairment score, with higher scores indicating worse reaction times and balance. Scores above 57 are indicative of impairment^24^ as they have previously been shown to be associated with blood-alcohol concentrations of 0.08%^25^. Cronbach’s alpha was 0.93 in the present study.

### Procedures

#### Recruitment

Participants were recruited using advertisements posted in cannabis dispensaries, at WSU, in the community, and on social media as well as by emailing cannabis users who had completed other THC lab studies. Prospective participants were informed that we were interested in understanding the acute effects of CBG and were directed to complete a brief online Qualtrics screening survey that included bot detection and contained questions probing the various inclusion/exclusion criteria.

#### Pre-testing session

Eligible participants were invited to a pre-testing Zoom session with either the principal investigator or RA to ensure they could access Zoom on a secure stable internet connection in a personal environment, obtain informed consent, download the DRUID app and complete the baseline trials, schedule their testing sessions, assign their ID code, and provide them with the contact information of a CBG producer who shipped color-coded vials of hemp-derived CBG and placebo directly to them. Participants were instructed to abstain from using any cannabis products including CBG for a minimum of 24 h prior to their testing session. This 24-h period was selected to avoid picking up any acute effects of cannabis while also ensuring participants were not experiencing withdrawal symptoms which typically peak 2–6 days after abstinence from cannabis^26^.

#### Testing sessions

**Figure 1:**
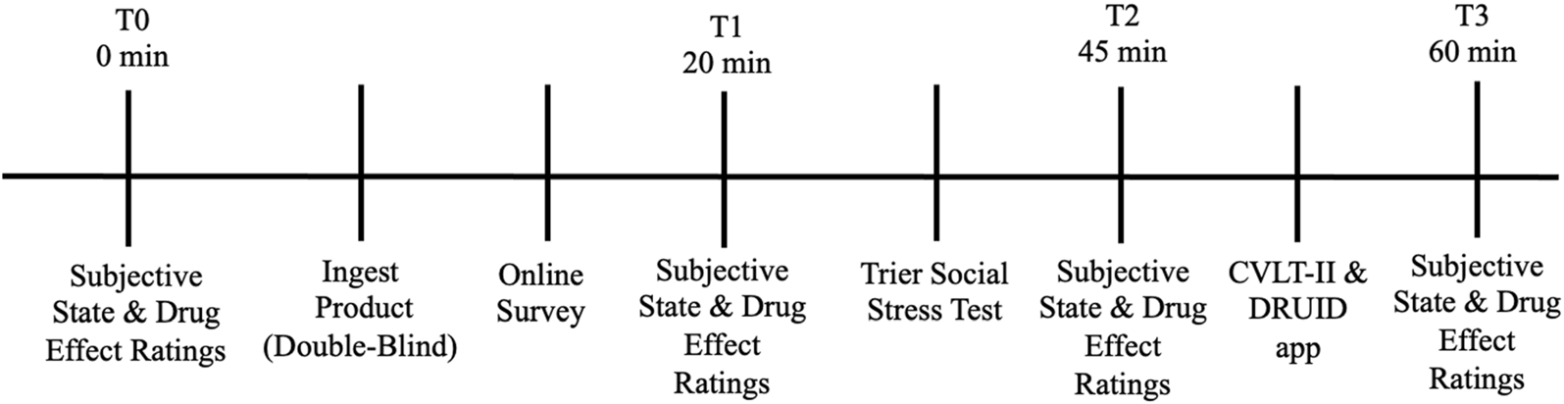
Overview of procedure for each testing session.

Approximately one-week after the pre-testing Zoom session participants met with a RA—who was blinded to the color-codes used for the drug and placebo—on Zoom for their first testing session. First, the RA confirmed that participants had abstained from use of cannabis and CBG for a minimum of 24 h prior to the testing session. As depicted in Fig. 1, participants then provided baseline (T0) ratings of their subjective state (anxiety, stress, mood, STAI state anxiety). Next, participants were instructed to ingest one of the color-coded vials. Half the participants were assigned odd ID codes and were randomly assigned to ingest the blue vial containing 20 mg CBG first and the other half were assigned even ID codes and were assigned to ingest the yellow vial containing 20 mg placebo first. To help disguise the taste, participants were instructed to mix the contents of the vial in a small glass of water prior to oral ingestion. After observing the participant ingest the product, the RA instructed them to complete an online survey that contained measures of their demographic characteristics; anxiety, depression, and stress levels; as well as cannabis and CBG use patterns.

After completing the online questionnaire, participants provided T1 ratings of their subjective state and subjective drug effects. The mean time between T0 and T1 ratings was 20 min. Participants subsequently completed the Trier Social Stress Test. Following the stress manipulation participants provided T2 ratings of their subjective state and subjective drug effects. The mean time between T0 and T2 ratings was approximately 45 min. Participants then completed the California Verbal Learning Test-II and the DRUID app. Finally, participants provided T3 ratings of their subjective state and subjective drug effects. The mean time between T0 and T3 ratings was approximately 60 min.

One-week after the first testing session, participants completed the second testing session. This session was identical to the first testing session except those participants who ingested the blue vial containing CBG in the first session, ingested the yellow vial containing placebo in the second session. Those who ingested the yellow vial containing placebo in the first session, ingested the blue vial containing CBG in the second session. Neither the participant nor the RA knew which color code corresponded to CBG and placebo.

### Data and statistical analysis

Percentages, means, and standard deviations were used to determine the demographic characteristics and cannabis use patterns of the sample. Mean DASS subscale scores, mean subjective state (mood, anxiety, stress) ratings, mean STAI state and trait anxiety scores, and mean subjective drug effect ratings were computed at baseline.

Change scores were created by subtracting baseline (T0) scores from T1, T2, and T3 scores for the subjective state ratings (anxiety, stress, mood), STAI state anxiety scores, and subjective drug effect ratings (dry eyes, dry mouth, sleepiness, appetite, heart palpitations/racing heart). Since intoxication scores were all 0 at baseline (T0) and drug effect and drug liking ratings were not obtained at baseline, raw post-drug administration ratings at T1, T2, and T3 were used in analyses of these secondary outcomes.

To determine the effects of CBG vs. placebo on subjective state ratings, STAI state anxiety scores, and subjective drug effect ratings (dry eyes, dry mouth, sleepiness, appetite, heart palpitations/racing heart) a series of 2 × 3 repeated-measures ANCOVAs were conducted with condition (CBG, placebo), and time (T1, T2, T3) as within-subjects factors, order of drug administration as a covariate, and changes (difference from T0 to T1, T2, T2) in ratings as the dependent variables.

To determine the effects of CBG vs. placebo on intoxication, drug effect, and drug liking ratings a series of 2 × 3 repeated-measures ANCOVAs were conducted with condition (CBG, placebo), and time (T1, T2, T3) as within-subjects factors, order of drug administration as a covariate, and ratings of each indicator of drug effects as the dependent variables.

To examine the acute effects of CBG on verbal memory, a 2 × 5 repeated-measures analysis of covariance (ANCOVA) was conducted with condition (CBG, placebo), and CVLT-II trial (Trial 1, Trial 2, Trial 3, Trial 1B, Short-Delay) as within-subjects factors, order of drug administration as a covariate, and number of words correctly recalled as dependent variable.

Finally, to assess the effects of CBG vs. placebo on impairment, a one-way repeated measures ANCOVA was conducted to compare DRUID scores at baseline, in the CBG condition, and in the placebo condition, while controlling for order.

Pairwise deletion was used to handle the small amount (< 1%) of missing data. Alpha was set to 0.05 and effect sizes of 0.01 are interpreted as small, 0.06 are considered medium, and 0.14 and above are considered large^27^. Data were analyzed using IBM SPSS v.27.

### Ethics approval

The study was approved by the Washington State University (WSU) Institutional Review Board (IRB).

## Results

### Participants

**Figure 2:**
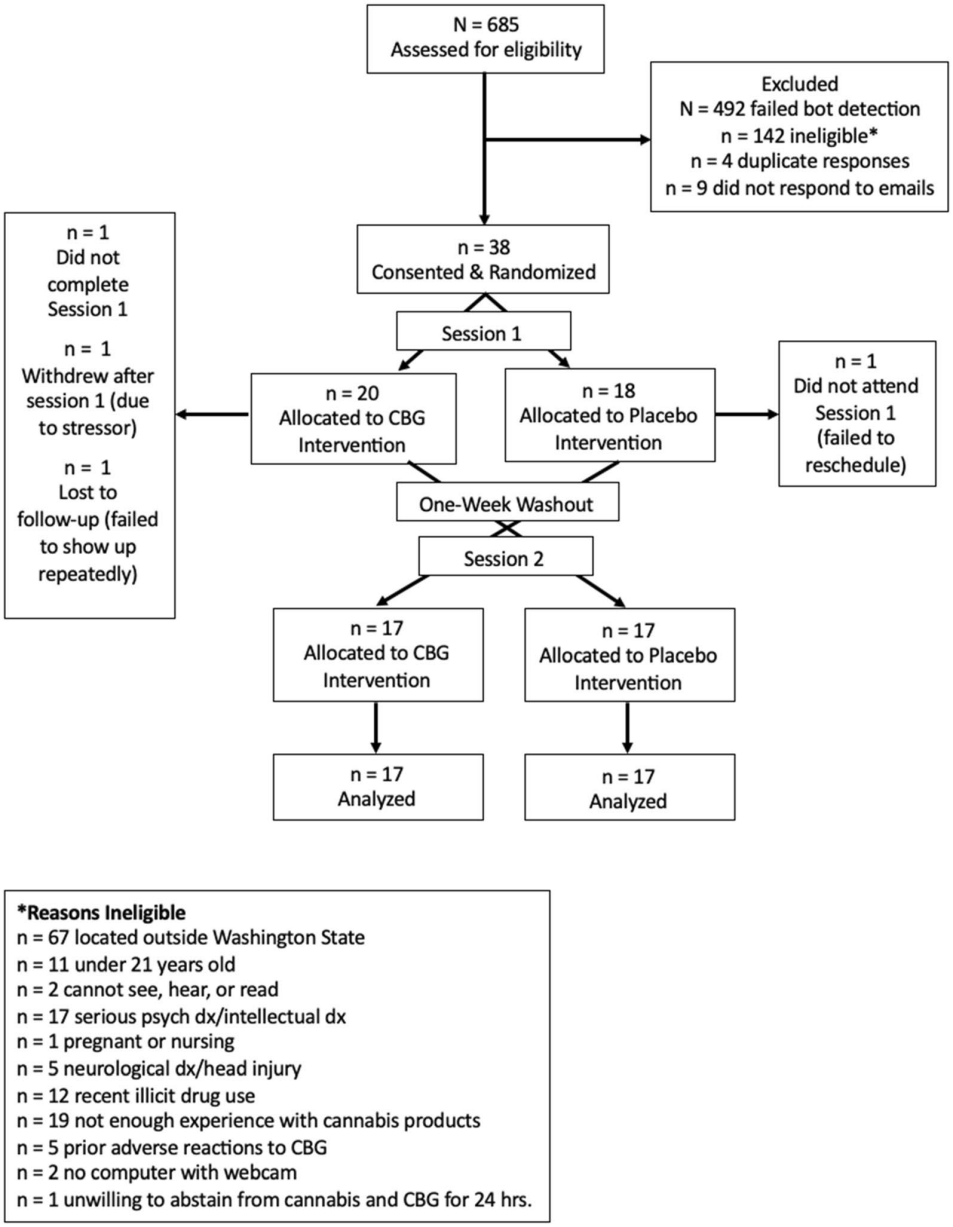
Consort flow diagram. Consort flow diagram showing the total number of people assessed for eligibility, randomized, tested, and analyzed with reasons for exclusions.

The sample of 34 eligible participants ranged in age from 21 to 60 (*M* = 30.06; *SD* = 10.50). Table 1 shows the remaining demographic characteristics of the sample. Figure 2 shows a Consort flow diagram of the number of participants screened, determined ineligible, and randomized.

**Table 1:** Demographic characteristics.

Gender	*n* (%)	Marital status	*n* (%)
Woman	18 (52.9)	Single/never married	26 (76.5)
Man	10 (29.4)	Married/domestic partnership	7 (20.6)
Non-binary/transgender	6 (17.6)	Divorced	1 (2.9)

Ethnicity	*n* (%)	Employment	*n* (%)
White	28 (82.4)	Full time	14 (41.2)
Black/African American	1 (2.9)	Part time	3 (8.8)
Hispanic/Latino	2 (5.9)	Unemployed	4 (11.8)
Asian	1 (2.9)	Student	13 (38.2)
Multiracial	2 (5.9)		

Education	*n* (%)	Income	*n* (%)
High school/GED	9 (26.5)	Less than $20,000	15 (44.1)
Technical school	3 (8.8)	$20,000–$40,000	7 (20.6)
Associate degree	6 (17.6)	$41,000–$60,000	7 (20.6)
Bachelor’s degree	12 (35.3)	$61,000–$80,000	5 (14.7)
Master’s degree	3 (8.8)		
Doctoral degree	1 (2.9)		

No participants endorsed being Native American, Pacific Islander, having less than a high school education, being widowed, divorced, retired, on disability, or earning a personal income higher than $80,000.

The majority of the sample reported no prior experience using CBG (*n* = 22, 64.7%). All participants endorsed prior experience using cannabis. Further, all participants indicated that they had abstained from cannabis use in the 24 h prior to each testing session with a mean period of abstinence of 96 h and a median of 48 h. Participants reported using cannabis 0–7 days of the past week (*M* = 3.62, *SD* = 2.45) and 1–31 days of the past month (*M* = 18.65, *SD* = 10.86). Participants reported using cannabis for 2 to 40 years in duration (*M* = 10.79, *SD* = 9.29) and being aged 8 to 29 when they first tried cannabis (*M* = 16.25, *SD* = 3.42). Most of the sample (*n* = 28, 82.4%) reported using cannabis for recreational purposes, with the remainder (*n* = 6; 17.6%) reporting using for recreational and medicinal purposes (none reported using for only medicinal purposes). The remaining cannabis and use patterns of the sample are provided in Table 2.

**Table 2:** Cannabis use patterns.

Cannabis use frequency	*n* (%)	Cannabis lifetime uses	*n* (%)
Once a month	1 (2.9)	11–50	3 (8.8)
2–3 times a month	3 (8.8)	51–100	2 (5.9)
Once a week	1 (2.9)	101–500	3 (8.8)
Twice a week	3 (8.8)	501–1000	3 (8.8)
3–4 times a week	7 (20.6)	1001–2000	7 (20.6)
5–6 times a week	6 (17.6)	2001–5000	7 (20.6)
Once a day	4 (11.8)	5001–10,000	1 (2.9)
More than once a day	9 (26.5)	More than 10,000	8 (23.5)

Primary method of administration	*n* (%)	Primary form of cannabis	*n* (%)
Joints	7 (20.6)	Flower	17 (50)
Hand pipe	3 (8.8)	Concentrates	9 (26.5)
Bong (water pipe)	5 (14.7)	Edibles	7 (20.6)
Vaporizer	12 (35.3)	Other	1 (2.9)
Edibles	7 (20.6)		

No participants reported using blunts or hookahs as their primary method of cannabis administration.

### Baseline measures

Participants’ baseline DASS or STAI subscale scores; subjective state ratings of mood, anxiety, and stress; ratings of intoxication and subjective drug effects prior to drug administration are provided in Table 3. Importantly, all participants gave baseline intoxication ratings of 0 in both conditions consistent with the required 24-h period of abstinence from cannabis and CBG.

**Table 3:** Baseline subjective state and drug effect scores.

Baseline (T0) ratings	Placebo	CBG
DASS	Mean (SD)	Mean (SD)
Depression	8.88 (10.81)	10.24 (10.59)
Anxiety	7.65 (6.60)	8.88 (7.74)
Stress	13.24 (10.48)	13.00 (10.44)
STAI		
State anxiety	37.18 (10.36)	36.85 (8.67)
Trait anxiety	44.42 (12.32)	44.29 (11.90)
Subjective state ratings		
Mood	6.76 (1.84)	7.18 (1.42)
Anxiety	2.91 (2.38)	3.59 (2.35)
Stress	3.59 (2.19)	3.56 (2.57)
Subjective drug effects		
Intoxication	0.00 (0.00)	0.00 (0.00)
Dry eyes	1.38 (1.99)	1.47 (2.16)
Dry mouth	1.44 (1.97)	1.59 (2.26)
Sleepy	3.41 (2.70)	2.68 (2.31)
Hunger	1.79 (1.89)	2.03 (2.14)
Racing heart	0.68 (1.61)	0.59 (1.08)

*DASS* depression, anxiety, stress scales, *STAI* state-trait anxiety inventory.

### Effects of CBG vs. placebo on subjective state ratings

**Figure 3:**
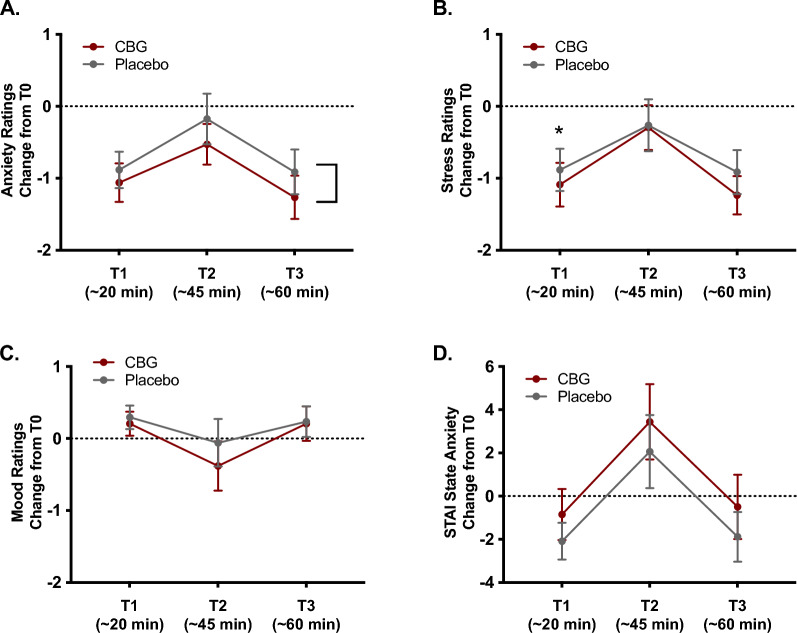
Changes in subjective state ratings following drug administration. Lines represent mean changes in subjective ratings of anxiety (**A**), stress (**B**), mood (**C**), and STAI state anxiety scores (**D**) from baseline (T0) to T1, T2, and T3. Error bars represent standard errors of the mean.

A 2 × 3 repeated-measures ANCOVA on changes in subjective anxiety ratings (changes from T0 to T1, T2, T3) revealed a moderately large-sized statistically significant main effect of condition, *F*(1, 32) = 4.88, *p* = 0.034, ${\upeta }_{\text{p}}^{2}$ = 0.132, and a large-sized statistically significant main effect of time, *F*(2, 64) = 7.76, *p* < 0.001, ${\upeta }_{\text{p}}^{2}$ = 0.195. The interaction between condition and time was small in magnitude and was not statistically significant, *F*(2, 64) = 1.56, *p* = 0.217, ${\upeta }_{\text{p}}^{2}$ = 0.047. As depicted in Fig. 3A, the main effect of condition reflects overall larger reductions in self-reported feelings of anxiety in the CBG condition compared to the placebo condition.

A 2 × 3 repeated measures ANCOVA on changes in subjective stress ratings revealed a large-sized statistically significant main effect of time, *F*(2, 64) = 8.20, *p* < 0.001, ${\upeta }_{\text{p}}^{2}$ = 0.204, and a small-sized main effect of condition that was not statistically significant, *F*(1, 32) = 1.19, *p* = 0.283, ${\upeta }_{\text{p}}^{2}$ = 0.036. This main effect of time was qualified by a moderately large-sized statistically significant interaction between condition and time, *F*(2, 64) = 4.90, *p* = 0.011, ${\upeta }_{\text{p}}^{2}$ = 0.133. Probing of this interaction revealed a moderately large-sized simple effect of condition on change in subjective stress ratings at T1, *F*(1, 32) = 5.02, *p* = 0.032, ${\upeta }_{\text{p}}^{2}$ = 0.136. In contrast, there were no significant differences in changes in stress ratings in the CBG and placebo conditions at T2, *F*(1, 32) = 0.31, *p* = 0.583, ${\upeta }_{\text{p}}^{2}$ = 0.010, or T3, *F*(1, 32) = 2.26, *p* = 0.142, ${\upeta }_{\text{p}}^{2}$ = 0.066 (see Fig. 3B).

The 2 × 3 repeated measures ANCOVA on changes in subjective mood ratings revealed no significant main effects of time, *F*(2, 64) = 2.56, *p* = 0.085, ${\upeta }_{\text{p}}^{2}$ = 0.074, or condition, *F*(1, 32) = 3.25, *p* = 0.081, ${\upeta }_{\text{p}}^{2}$ = 0.092, and no condition x time interaction, *F*(2, 64) = 2.27, *p* = 0.111, ${\upeta }_{\text{p}}^{2}$ = 0.066 (Fig. 3C).

The 2 × 3 repeated-measures ANCOVA on changes in STAI state anxiety total scores (changes from T0 to T1, T2, T3) revealed no significant main effect of condition, *F*(1, 32) = 2.47, *p* = 0.126, ${\upeta }_{\text{p}}^{2}$ = 0.072, but a large-sized statistically significant main effect of time, *F*(2, 64) = 7.54, *p* = 0.001, ${\upeta }_{\text{p}}^{2}$ = 0.191, that was qualified by a medium sized statistically significant interaction between condition and time, *F*(2, 64) = 3.68, *p* = 0.031, ${\upeta }_{\text{p}}^{2}$ = 0.103. Nevertheless, probing of this interaction revealed no significant effects of drug condition on changes in STAI state anxiety scores at T1, *F*(1, 32) = 0.09, *p* = 0.764, ${\upeta }_{\text{p}}^{2}$ = 0.003; T2, *F*(1, 32) = 3.10, *p* = 0.088, ${\upeta }_{\text{p}}^{2}$ = 0.088; or T3, *F*(1, 32) = 3.74, *p* = 0.062, ${\upeta }_{\text{p}}^{2}$ = 0.105 (Fig. 3D).

Supplemental Fig. S1 further depicts raw score (rather than change score) ratings of anxiety, stress, mood and STAI anxiety scores from T0, T1, T2, and T3.

### Effects of CBG vs. placebo on subjective drug effect ratings

**Figure 4:**
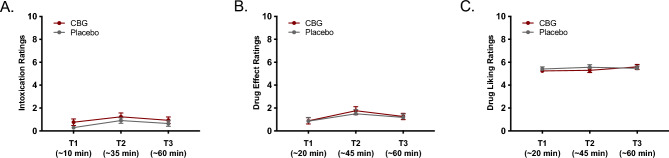
Intoxication, drug effect, and drug liking ratings following drug administration. Lines represent mean self-reported intoxication (**A**), drug effect (**B**), drug liking (**C**) ratings following drug/placebo administration. Error bars represent standard errors of the mean.

There were no significant effects of condition [*F*(1, 32) = 3.72, *p* = 0.063, ${\upeta }_{\text{p}}^{2}$ = 0.104], time [*F*(2, 64) = 0.32, *p* = 0.724, ${\upeta }_{\text{p}}^{2}$ = 0.010], or condition x time interaction [*F*(2, 64) = 0.34, *p* = 0.714, ${\upeta }_{\text{p}}^{2}$ = 0.010] on subjective ratings of intoxication. There were no significant effects of condition [*F*(2, 64) = 2.99, *p* = 0.093, ${\upeta }_{\text{p}}^{2}$ = 0.085], time [*F*(1, 32) = 1.71, *p* = 0.188, ${\upeta }_{\text{p}}^{2}$ = 0.051], or condition x time interaction [*F*(2, 64) = 0.04, *p* = 0.958, ${\upeta }_{\text{p}}^{2}$ = 0.001] on drug liking ratings. There were no significant effects of condition [*F*(2, 64) = 0.73, *p* = 0.789, ${\upeta }_{\text{p}}^{2}$ = 0.002], time [*F*(1, 32) = 1.08, *p* = 0.345, ${\upeta }_{\text{p}}^{2}$ = 0.033], or condition x time interaction [*F*(2, 64) = 0.25, *p* = 0.779, ${\upeta }_{\text{p}}^{2}$ = 0.008] on drug liking ratings. Moreover, intoxication (Fig. 4A) and drug effect (Fig. 4B) ratings remained low (under 2 out of 10) in both conditions at all timepoints, and drug liking ratings (Fig. 4C) remained neutral (5 out of 10) in both conditions at all timepoints.

**Figure 5:**
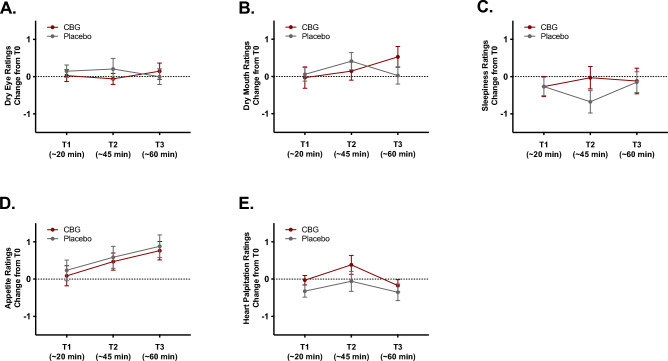
Subjective drug effect ratings following drug administration. Lines represent self-reported changes (from T0) in dry eyes (**D**), dry mouth (**E**), sleepiness (**F**), appetite (**G**), and heart palpitations (**H**) following drug administration. Error bars represent standard errors of the mean.

There were no significant effects of condition [*F*(1, 32) = 0.22, *p* = 0.640, ${\upeta }_{\text{p}}^{2}$ = 0.007], time [*F*(2, 64) = 0.17, *p* = 0.847, ${\upeta }_{\text{p}}^{2}$ = 0.005], or condition x time interaction [*F*(2, 64) = 0.68, *p* = 0.508, ${\upeta }_{\text{p}}^{2}$ = 0.021] on changes in dry eye ratings (see Fig. 5A). There were no significant effects of condition [*F*(1, 32) = 0.59, *p* = 0.448, ${\upeta }_{\text{p}}^{2}$ = 0.018], time [*F*(2, 64) = 1.85, *p* = 0.166, ${\upeta }_{\text{p}}^{2}$ = 0.055], or condition x time interaction [*F*(2, 64) = 1.86, *p* = 0.164, ${\upeta }_{\text{p}}^{2}$ = 0.055] on changes in dry mouth ratings (see Fig. 5B). There were no significant effects of condition [*F*(1, 32) = 0.03, *p* = 0.863, ${\upeta }_{\text{p}}^{2}$ = 0.001], time [*F*(2, 64) = 1.35, *p* = 0.267, ${\upeta }_{\text{p}}^{2}$ = 0.040], or condition x time interaction [*F*(2, 64) = 2.39, *p* = 0.100, ${\upeta }_{\text{p}}^{2}$ = 0.069] on changes in sleepiness ratings (see Fig. 5C). There was a significant main effect of time [*F*(2, 64) = 7.05, *p* = 0.002, ${\upeta }_{\text{p}}^{2}$ = 0.181], but no significant effect of condition [*F*(1, 32) = 0.15, *p* = 0.701, ${\upeta }_{\text{p}}^{2}$ = 0.005], or condition x time interaction [*F*(2, 64) = 0.28, *p* = 0.754, ${\upeta }_{\text{p}}^{2}$ = 0.009] on changes in appetite ratings. As depicted in Fig. 5D, the main effect of time simply reflects an increase in appetite over time in both conditions. There was a significant main effect of time [*F*(2, 64) = 4.19, *p* = 0.020, ${\upeta }_{\text{p}}^{2}$ = 0.116], but no significant effect of condition [*F*(1, 32) = 0.18, *p* = 0.674, ${\upeta }_{\text{p}}^{2}$ = 0.006], or condition x time interaction [*F*(2, 64) = 0.17, *p* = 0.843, ${\upeta }_{\text{p}}^{2}$ = 0.005] on changes in heart palpitation/racing heart ratings. As depicted in Fig. 5E, the main effect of time simply reflects a slight increase in heart palpitations following the Trier Social Stress Test (from T1 to T2).

### Verbal memory

**Figure 6:**
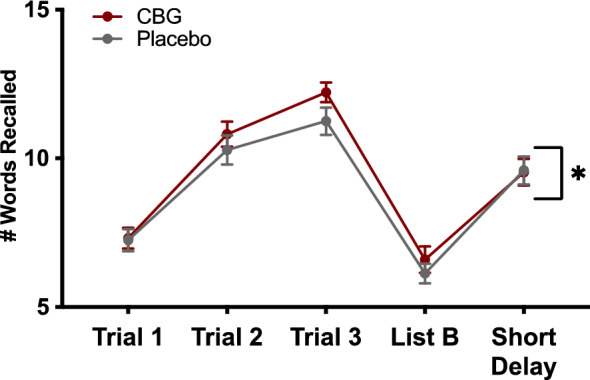
Effects of CBG vs placebo memory. Lines represent mean number of words recalled in Trial 1, Trial 2, Trial 3, List B, and the Short Delay recall trial of the CVLT-II. Error bars show standard errors of the mean.

A 2 × 5 repeated measures ANCOVA with condition (CBG, placebo) and trial as within-subjects factors and order as a covariate revealed a moderate-sized effect of condition that was statistically significant, *F*(1, 30) = 4.17, *p* = 0.050, ${\upeta }_{\text{p}}^{2}$ = 0.122 and a large-sized effect of time that was statistically significant, *F*(4, 120) = 70.14, *p* < 0.001, ${\upeta }_{\text{p}}^{2}$ = 0.700. The interaction between condition and time was small and not statistically significant, *F*(4, 120) = 0.38, *p* = 0.820, ${\upeta }_{\text{p}}^{2}$ = 0.013. As depicted in Fig. 6, verbal memory test performance was significantly better in the CBG condition.

### DRUID impairment

**Figure 7:**
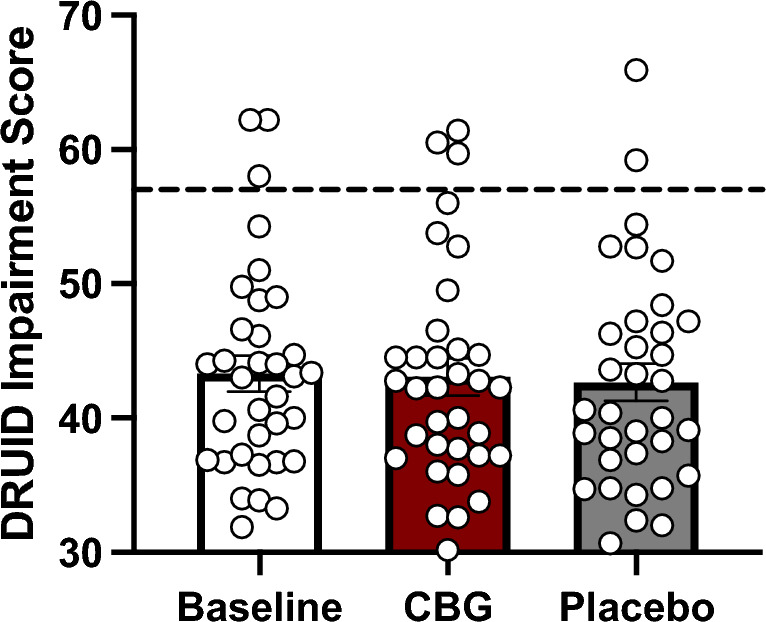
Effects of CBG vs placebo on impairment. Bars represent mean global impairment scores at Baseline (T0), following ingesting of CBG and Placebo. Circles represent individual scores in each condition. Error bars represent standard errors of the mean. The dashed line represents a score of 57 which indicates significant impairment.

The one-way repeated measures ANCOVA on DRUID impairment scores revealed a small sized effect of condition that was not statistically significant, *F*(2, 64) = 0.76, *p* = 0.474, ${\upeta }_{\text{p}}^{2}$ = 0.023 (see Fig. 7).

## Discussion

The rapid proliferation of the legal cannabis market has provoked producers to cultivate an array of novel products to satisfy consumers' growing interests, including products dominant in CBG. Pre-clinical research indicates CBG may have a broad spectrum of therapeutic effects that have recently been supported by self-report data from a large sample of CBG users^12^. The present study represents the first human clinical trial to examine the acute effects of CBG on anxiety, stress, and mood. Results indicate that CBG reduces global feelings of anxiety and stress and that it may enhance memory in the absence of intoxication, impairment, or subjective drug effects.

Most notably, there was a significant main effect of CBG on subjective state ratings of anxiety. Specifically, there was a mean decrease in anxiety ratings of 0.95 on a 10-point scale which represents a 26.5% reduction in the already low levels of baseline anxiety (*M* = 3.59 on a 0–10 VAS) in the CBG condition. In contrast there was a 0.66-point reduction in anxiety ratings in the placebo condition (which represents a 22.5% reduction in anxiety in the placebo condition). These findings are in line with our survey findings wherein 51% of CBG users reported using CBG to manage anxiety and 78% claimed it was more effective than conventional anxiety medications^12^. These effects may stem from CBG’s effects on 5-HT_1A_^28,29^ and/or GABA^30^. Nevertheless, enthusiasm for these findings is dampened by the lack of significant effect of CBG on STAI state anxiety ratings. This pattern of findings may reflect a tendency for CBG to reduce the global impression of feelings of anxiety, rather than affecting the specific sub-components of anxiety tapped by the STAI (e.g., jittery, confused, indecisive) and further indicate that replication of these findings is necessary.

Consistent with these effects, there was also a significant effect of CBG on subjective stress ratings at T1 (prior to the stressor). While our power analysis indicated we were sufficiently powered to detect medium-sized effects, a larger sample size and/or higher dose may be needed to robustly detect acute effects of CBG on subjective stress ratings. Our use of a field trial precluded our ability to examine physiological indicators of stress (e.g., cortisol, electrodermal activity, alpha-amylase) but we plan to conduct a follow-up laboratory study which will include such measures. As no human clinical trials with CBG had been published at the time we commenced the study our dose selection was guided by anecdotal reports of people using the same CBG tincture, observations that CBG is commonly sold in 10 mg units, as well as by common doses of CBG reported in clinical observations^31,32^. However, given recent clinical trials using larger doses (25 mg^12^ and 50 mg^13^), our dose may have been somewhat conservative.

Our prior survey study showed that over 30% of CBG users were self-medicating for depression and 80% reported CBG is more effective than conventional antidepressants^12^ which is consistent with preclinical research demonstrating that CBG has antidepressant-like effects in a rodent tail suspension model^10^. Nonetheless, results from the present clinical trial failed to support our hypothesis that CBG would enhance mood. It is possible that our sensitivity to detect such effects was reduced by the administration of a single-item indicator of mood to a non-clinical sample. Indeed, the overall mean baseline depression levels on the DASS indicates that the sample was in the normal to mild range of depression^19^ and the overall baseline mood ratings suggest participants were in a positive mood state prior to drug administration. Future research employing a more comprehensive measure of depression and a larger sample of clinical patients with higher baseline depression is needed to attempt to reconcile these apparently contradictory findings.

CBG did not produce cognitive or motor impairments on the DRUID app. Prior placebo-controlled research reported DRUID scores around 57 following oral administration of 25 mg of THC and vaporized administration of 20 mg of THC^24^ which is on par with the threshold of 57 for detecting significant impairment using this app^25^. In contrast, participants’ mean DRUID score following administration of CBG was much lower (43.1) and was remarkably consistent with the mean DRUID score detected at baseline and in the placebo condition. As shown in Fig. 7, a small number of people surpassed the 57-score threshold for impairment at baseline and in both the CBG and placebo conditions which is likely attributable to preexisting conditions (e.g., one participant reported issues with balance, another had a leg injury).

One of the most robust detrimental effects of THC is on verbal memory^33^ which guided our decision to include a test of verbal memory in the present study. We hypothesized that CBG would not impair memory, but our finding that CBG significantly *enhanced* verbal memory was unexpected. Inspection of Fig. 6 shows that the effect was most pronounced on trials 2 & 3 with participants in the CBG condition recalling on average 0.5 words more on trial 2 and 1-word more on trial 3, than they recalled in the placebo condition. Trials 2 & 3 represents learning trials which indicates that CBG may enhance learning. Nevertheless, these surprising findings warrant further corroboration. Future studies attempting to replicate these findings should include tests of verbal memory with additional learning trials, as well as other tests of memory (e.g., working memory) and cognition (e.g., executive functioning, attention) to further elucidate the effects of CBG on cognition. It would also be interesting to examine whether CBG might offset the detrimental effects of THC on verbal memory, as CBD was initially purported to do^34,35^, until multiple attempts to replicate those findings failed^36–38^.

Consistent with the lack of impairment detected in the CBG condition, participants reported no intoxicating effects of CBG. The overall mean intoxication rating (combined across the three time points) was less than 1 on a 0–10 VAS in both the CBG and placebo conditions. Similarly, overall mean ratings of subjective drug effects were low in both conditions and drug liking effects were neutral in both conditions at all timepoints suggesting a low potential for abuse. Further, there were no significant effects of CBG on changes in dry eyes, dry mouth, sleepiness, appetite, or heart palpitation ratings, with ratings changing less than 1-point from baseline over the course of the experiment. This suggests that CBG was well tolerated and did not elicit adverse acute effects that are typically associated with THC administration. Our decision to assess effects on these outcomes was informed by our prior survey study^12^ that revealed that while the plurality (44%) indicated CBG has no side effects, small percentages of participants endorsed experiencing dry eyes, dry mouth, sleepiness, and increased appetite after using CBG-dominant cannabis, perhaps because some were using cannabis containing both CBG and THC. It is also important to note that five individuals who completed our screening survey indicated they had previously experienced a severe adverse reaction to CBG. While the extent to which these events were triggered by CBG taken in combination with THC are unknown, they suggest we should be cautious in our interpretation of these null findings. Additional caution should be taken when interpreting results from the present study because we conducted this study remotely via Zoom so there were no physical exams or assessments of vital signs, participants were limited to reporting on a set of pre-determined potential adverse events and were not asked about these in an open-ended manner, these assessments were limited to the first 20–60 min after dosing and only a single, relatively modest dose was assessed which may have missed some of the peak effects^14^. As such, these results cannot be extrapolated to other potential adverse effects, later timepoints, higher doses, or repeated/chronic dosing. Future research should examine a broader array of potential drug effects and side effects using physiological measures (heart rate monitors, blood pressure monitors) across longer periods of time prior to reaching strong conclusions that CBG is safe and not associated with side effects.

While our use of a gold-standard double-blind, placebo-controlled cross-over design increases internal validity and our use of Zoom to naturalistically test participants in their home environments enhances ecological validity, the study is not without its limitations. These limitations include our use of a non-clinical sample of cannabis users, a lack of correction for multiple statistical comparisons, modest effects of the stress manipulation, as well as the use of a relatively modest dose and the early timing of assessments. Specifically, as a prudent first step we recruited a moderate-sized community sample rather than a clinical sample of patients with anxiety or depression. A larger sized clinical sample may have increased our sensitivity to detect significant effects of CBG on mood, stress, and state anxiety but may have also increased risks as potential drug interactions with SSRIs are unknown. We also used a sample of experienced cannabis users (for ethical reasons) and as such must be very cautious in generalizing the findings to cannabis naïve individuals. Although potential interactions between THC and CBG are unknown, our use of experienced cannabis users may have also diminished our sensitivity to detect effects due to a general level of tolerance with cannabinoids. We also did not correct for the multiple statistical comparisons that were conducted as the study was not powered to do so. As such, some findings may represent Type I errors and replication is required to corroborate them. Further, while the acute stress manipulation significantly increased stress from T1 to T2, average subjective stress ratings remained below the baseline (T0) after the stressor. While the online version of the TSST has been previously validated^22^, a stress manipulation performed in the lab (rather than in participants’ comfortable home environments via Zoom) may have produce larger increases in subjective stress. However, the relatively modest increases in subjective stress following the TSST may also reflect our use of a sample of experienced cannabis users, as prior research has shown that cannabis users demonstrate blunted cortisol and subjective stress responses to acute stressors even in a laboratory environment^39^.

At the time we commenced the study there had been no published clinical trials of CBG in humans, as such, we had little to guide us on the most appropriate dose or timing of assessments. As indicated previously, our dose selection was guided by anecdotal evidence from people using this tincture, standard doses of CBG in marketed products, and clinical observations of efficacy at similar doses^31,32^. However, given recent clinical trials using larger doses of CBG (25 mg^12^ and 50 mg^13^) our dose may have been too conservative to reveal optimal effects. Repeated dosing may have also increased potential effects on stress, anxiety, and mood. Further, while recent data from a human pharmacokinetic study of CBG^14^ demonstrated rises in plasma concentrations as early as 20 min after oral administration, these concentrations peaked from 45 min to nearly 2 h. (depending on dietary fat). As such our assessments from 20 to 60 min after dosing may have failed to capture peak effects. Additionally, we did not restrict participant’s diets in anyway, but recent evidence indicates that eating a meal high in dietary fat prior to ingestion of CBG increases plasma levels of CBG^14^. Our failure to control diet may have resulted in random variability in the potency of the product that could have contributed to random error that further diminished sensitivity to detect significant effects.

In conclusion, results of this double-blind, placebo-controlled, cross-over field trial indicate that 20 mg of hemp-derived CBG reduces subjective ratings of anxiety and stress in healthy cannabis-using adults in the absence of motor or cognitive impairment, intoxication, or other subjective drug effects (e.g., heart palpitations, dry mouth). Additional research is needed to corroborate these novel findings as well as to extend them to a clinical population of patients with anxiety disorders.

### Supplementary Information

Supplementary Information 1.

Supplementary Information 2.

Supplementary Figure 1.

## Data Availability

Data are available upon reasonable request to carrie.cuttler@wsu.edu.
