# Supplementary material for: Acute effects of cannabigerol on anxiety, stress, and mood: a double-blind, placebo-controlled, crossover, field trial
Source: Sci Rep. 2024 Jul 13;14:16163. doi: 10.1038/s41598-024-66879-0 (PMC11246434; doi:10.1038/s41598-024-66879-0)
Supplement: Supplementary file 1 — Supplementary Information 1. [file 41598_2024_66879_MOESM1_ESM.pdf]

Certificate of Analysis

CBG Cultivar

LeBlanc CNE

Test Result UID:	ANL0014562
Washington State Lot Inventory ID:	
Washington State Lab Inventory ID:	
Date Tested:	09/12/2018
Serving Size:	=

Photographs

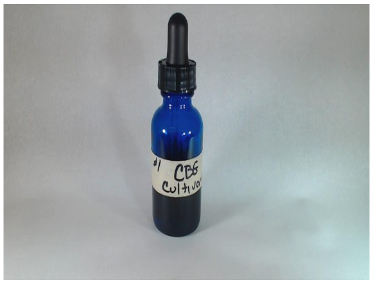

Summary

Cannabinoids:	THC Total:	< 0.01 mg/g<br>--	CBD Total:	< 0.01 mg/g<br>--
Terpene Total:	Not Tested<br>Not Tested			
Microbial:	Not Tested			
Pesticides:	Not Tested			
Heavy Metals:	Not Tested			
Water Activity:	Not Tested			

Mycotoxins (Method: ELISA)

Aflatoxins Total:	Not Tested	Not Tested	Not Tested
Ochratoxin A Total:	Not Tested	Not Tested	

Certificate of Analysis

CBG Cultivar

LeBlanc CNE

Test Result UID:	ANL0014562
Washington State Lot Inventory ID:	
Washington State Lab Inventory ID:	
Date Tested:	09/12/2018
Serving Size:	=

Potency Profile (Method: HPLC-DAD)

CBG-A	0.89 mg/g	--
CBG	10.04 mg/g	--
**CBG TOTAL** (CBG-A * 0.878 + CBG)[1]	10.82 mg/g	--
Δ9-THC-A	< 0.01 mg/g	--
Δ9-THC	< 0.01 mg/g	--
Δ9-THCV	< 0.01 mg/g	--
Δ8-THC	< 0.01 mg/g	--
CBN	< 0.01 mg/g	--
**THC-TOTAL** (THC-A * 0.877 + THC)[1]	< 0.01 mg/g	--
CBD-A	< 0.01 mg/g	--
CBD	< 0.01 mg/g	--
CBDV-A	< 0.01 mg/g	--
CBDV	< 0.01 mg/g	--
**CBD-TOTAL** (CBD-A * 0.877 + CBD)[1]	< 0.01 mg/g	--
CBC	< 0.01 mg/g	--
**ACTIVATED-TOTAL** (Δ9THC + 9-THCV + Δ8THC + CBN + CBD + CBDV + CBG + CBC)[2]	10.04 mg/g	--
**TOTAL DETECTED CANNABINOIDS** (CBDV TOTAL + THC TOTAL + CBD TOTAL + CBG TOTAL + D8THC + CBN + CBC + THCV)	10.82 mg/g	--

1 - Cannabinoid totals are adjusted to account for the decarboxylation of the cannabinoid acids. The reported total is the amount of the activated cannabinoid that would be if all of the carboxylic acid has been removed through decarboxylation.

2 - Cannabinoids that have been activated through decarboxylation (curing/storage of flowers, or heating/cooking of edibles, tinctures, & concentrates)

Certificate of Analysis

CBG Cultivar LeBlanc CNE

Test Result UID:	ANL0014562
Washington State Lot Inventory ID:	
Washington State Lab Inventory ID:	
Date Tested:	09/12/2018
Serving Size:	=

Analytical 360, LLC certifies that the results presented on the previous 3 pages are true and correct to the best of our knowledge. These results relate only to the sample provided by the client to Analytical 360, LLC.

Approved by: Paul D. Matthews, Ph.D

Lab Director/Chief Science Officer

UBI: 603120434
Lab: 0004

Reference Lab:

Analytical 360 subcontracts the following assays:

Mycotoxins and Water Activity performed by Capitol Analysis (Lab #0022)

Labtech Notes

- None
