# Supplementary material for: Acute effects of cannabigerol on anxiety, stress, and mood: a double-blind, placebo-controlled, crossover, field trial
Source: Sci Rep. 2024 Jul 13;14:16163. doi: 10.1038/s41598-024-66879-0 (PMC11246434; doi:10.1038/s41598-024-66879-0)
Supplement: Supplementary file 2 — Supplementary Information 2. [file 41598_2024_66879_MOESM2_ESM.pdf]

Certificate of Analysis

CBG Trial

LeBlanc CNE

Test Result UID:	ANL0016149
Washington State Lot Inventory ID:	
Washington State Lab Inventory ID:	
Date Tested:	10/26/2018

Photographs

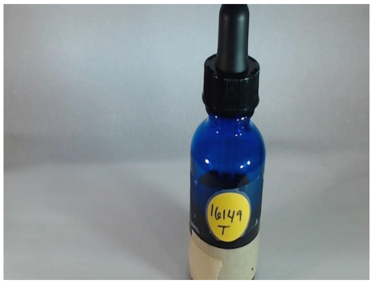

Summary

Cannabinoids:	THC Total:	Not Tested	CBD Total:	Not Tested
Terpene Total:	0.05 %		Solvent Total:	Not Tested
Microbial:	Not Tested		Pesticides:	Not Tested
Heavy Metals:	Not Tested			
Water Activity:	Not Tested	Not Tested		

Mycotoxins (Method: ELISA)

Aflatoxins Total:	Not Tested	Not Tested	Not Tested
Ochratoxin A Total:	Not Tested	Not Tested	

Certificate of Analysis

CBG Trial

LeBlanc CNE

Test Result UID: ANL0016149
Washington State Lot Inventory ID:
Washington State Lab Inventory ID:
Date Tested: 10/26/2018

Terpene Profile (Method: HS-GC-FID)

Alpha Pinene	< 0.01 mg/g	< 0.01 %
Beta Pinene	< 0.01 mg/g	< 0.01 %
Myrcene	< 0.01 mg/g	< 0.01 %
Ocimene	< 0.01 mg/g	< 0.01 %
Limonene	< 0.01 mg/g	< 0.01 %
Terpinolene	< 0.01 mg/g	< 0.01 %
Linalool	< 0.01 mg/g	< 0.01 %
Caryophyllene	0.35 mg/g	0.03 %
Humulene	0.16 mg/g	0.02 %
**Terpene Total**	0.51 mg/g	0.05 %

Certificate of Analysis

CBG Trial

LeBlanc CNE

Test Result UID:	ANL0016149
Washington State Lot Inventory ID:	
Washington State Lab Inventory ID:	
Date Tested:	10/26/2018

Analytical 360, LLC certifies that the results presented on the previous 3 pages are true and correct to the best of our knowledge. These results relate only to the sample provided by the client to Analytical 360, LLC.

Approved by: Paul D. Matthews, Ph.D

Lab Director/Chief Science Officer

UBI: 603120434
Lab: 0004

Reference Lab:

Analytical 360 subcontracts the following assays:

Mycotoxins and Water Activity performed by Capitol Analysis (Lab #0022)

Labtech Notes

- None
