# Supplementary material for: Acute effects of cannabigerol on anxiety, stress, and mood: a double-blind, placebo-controlled, crossover, field trial
Source: Sci Rep. 2024 Jul 13;14:16163. doi: 10.1038/s41598-024-66879-0 (PMC11246434; doi:10.1038/s41598-024-66879-0)
Supplement: Supplementary file 3 — Supplementary Figure 1. [file 41598_2024_66879_MOESM3_ESM.docx]

**Supplemental Figure 1**

**Raw Score Subjective State Ratings Before (T0) and After (T1-T3) Drug Administration**

Figure Caption: Lines represent mean subjective ratings of anxiety (panel A), stress (panel B), mood (panel C), and STAI state anxiety scores (panel D) in raw score form at baseline (T0), T1, T2, and T3. Error bars represent standard errors of the mean.
